# Acrylamide Exposure Destroys the Distribution and Functions of Organelles in Mouse Oocytes

**DOI:** 10.3389/fcell.2022.834964

**Published:** 2022-02-28

**Authors:** Chao-Ying Zhao, Lin-Lin Hu, Chun-Hua Xing, Xiang Lu, Shao-Chen Sun, Yu-Xia Wei, Yan-Ping Ren

**Affiliations:** ^1^ College of Basic Medical Sciences, Zunyi Medical University, Zunyi, China; ^2^ College of Animal Science and Technology, Nanjing Agricultural University, Nanjing, China; ^3^ Reproductive Medicine Center, The Affiliated Hospital of Youjiang Medical University for Nationalities, Baise, China

**Keywords:** acrylamide, oocyte, mitochondria, Golgi apparatus, lysosome

## Abstract

Acrylamide (ACR) is a common industrial ingredient which is also found in foods that are cooked at high temperatures. ACR has been shown to have multiple toxicities including reproductive toxicity. Previous studies reported that ACR caused oocyte maturation defects through the induction of apoptosis and oxidative stress. In the present study, we showed that ACR exposure affected oocyte organelle functions, which might be the reason for oocyte toxicity. We found that exposure to 5 mM ACR reduced oocyte maturation. ACR caused abnormal mitochondrial distribution away from spindle periphery and reduced mitochondrial membrane potential. Further analysis showed that ACR exposure reduced the fluorescence intensity of Rps3 and abnormal distribution of the endoplasmic reticulum, indicating that ACR affected protein synthesis and modification in mouse oocytes. We found the negative effects of ACR on the distribution of the Golgi apparatus; in addition, fluorescence intensity of vesicle transporter Rab8A decreased, suggesting the decrease in protein transport capacity of oocytes. Furthermore, the simultaneous increase in lysosomes and LAMP2 fluorescence intensity was also observed, suggesting that ACR affected protein degradation in oocytes. In conclusion, our results indicated that ACR exposure disrupted the distribution and functions of organelles, which further affected oocyte developmental competence in mice.

## Introduction

Acrylamide (ACR) is widely used in industrial production since the end of the 19th century (Semla et al., 2017). ACR was found in fried food until the early 2000s (Mojska and Gielecinska, 2013), and multiple studies determined the widespread existence of ACR in foods (Busova et al., 2020a). ACR is a kind of water-soluble chemical (Kumar et al., 2018), which can enter human organs and tissues through food and drinking water, causing toxic effects on multiple organs (Busova et al., 2020b) including reproductive systems (Bin-Jumah et al., 2021). Previous results show that ACR can cause peroxidation of cholinergic transmitter in the nervous system and decrease the transmission ability, leading to the damage of the nervous system (Kopanska et al., 2018). ACR can reduce the expression of catalase in the process of metabolism *in vivo*, thus increasing the toxicity of the liver and kidney (Hou et al., 2021). In addition, ACR enters the body through the respiratory tract, inducing oxidative stress on lung epithelial BEAS-2B cells, which leads to morphological changes and even apoptosis (Kacar et al., 2019). After oral administration of ACR to female SD rats, the pancreatic cells of rats can be damaged, resulting in the decrease in the insulin level in plasma (Yue et al., 2019). Furthermore, ACR can increase the risk of ovarian cancer by altering sex hormone levels (Hogervorst et al., 2017), and excessive ACR intake by pregnant women can inhibit the normal fetal growth (Hogervorst et al., 2021). Recent studies indicated that ACR exposure could disrupt oocyte maturation ability through its effects on oxidative stress and apoptosis in a mouse model (Aras et al., 2017), and oral ACR can reduce the ovarian weight and the number of GV oocytes in mice (Duan et al., 2015).

Oocyte maturation needs to undergo two asymmetric meiosis (Sen and Caiazza, 2013), and the mature ability of oocytes is closely related to successful fertilization and healthy development of embryos, which is one of the important markers for oocyte quality. During oocyte maturation, organelles are important in this process. The mitochondrion is the organelle that provides energy for cell life activities (Annesley and Fisher, 2019), and the ribosome is the only organelle that synthesizes proteins (Finkelstein et al., 2018). In addition, the endoplasmic reticulum includes rough endoplasmic reticulum and smooth endoplasmic reticulum (Sanvictores and Davis, 2021), and the rough endoplasmic reticulum is attached to ribosomes for protein processing and modification (Mandon et al., 2013), then passes it to the Golgi apparatus (Al-Qudah and Al-Dwairi, 2016), while the smooth endoplasmic reticulum has no ribosome attachment (Sanvictores and Davis, 2021), and it generates most lipids needed by the body (Sanvictores and Davis, 2021). The Golgi apparatus helps protein transported by the endoplasmic reticulum to further mature and form functional protein (Nakamura et al., 2012), and vesicles transport it to the position it functions (Bexiga and Simpson, 2013). Also, lysosomes digest excessive macromolecular substances and metabolites in the cell into small molecules for use by the cell itself or excrete it for use by other cells (Ferguson, 2019). Previous studies have also shown that different organelle dysfunction can lead to oocyte maturation defects. Treatment of mouse oocytes with hydrogen peroxide induced mitochondrial dysfunction *in vitro*, which affects the development of mouse embryos (Usama et al., 2020). If the function of ribosomes is damaged during ovary organization and oogenesis in phreodrilid clitellate, it can bring about oocyte dysfunction or abnormal fertilization (Witek et al., 2020). The expression of endoplasmic reticulum stress-related proteins in mouse oocytes increased in obese mice, which led to the decrease in mouse oocyte quality and embryo development (Rao et al., 2020). The disruption of cis-Golgi apparatus marker protein GM130 causes the decrease in the polar body rate and the quality of mouse oocytes (2015). The increased expression of lysosome marker protein LAMP2 in aged mice induces the decline of fertilization ability of mouse oocytes (Mcginnis et al., 2014).

In the present study, the effects of ACR on the distribution and function of mitochondria, ribosomes, endoplasmic reticulum, Golgi apparatus, and lysosomes in oocytes were detected, which was to determine the possible causes of ACR on the quality of oocytes from the organelle aspect. Our results suggested that ACR could cause the abnormal distribution of organelles in oocytes, indicating that ACR disrupted the process of synthesis, processing, modification, and degradation of protein.

## Materials and Methods

### Acquisition and Cultivation of Mouse Oocytes

This study followed the guidelines of the Animal Experiment and Research Committee of Nanjing Agricultural University. The experimental sample is the oocytes of 4-week-old female mice. The mice were kept in a 25 °C constant temperature room alternately day and night and were provided with enough water and feed. GV stage oocytes obtained from mouse ovaries were washed with M2 culture medium (Sigma, MO, United States) to obtain denuded oocytes. Then, we cultured oocytes in M2 culture medium covered with paraffin oil and incubated at 37°C and 5% CO_2_ for 8.5 h to obtain MI stage oocytes, and we obtained MII stage oocytes after 12 h of incubation.

### Treatment of Oocytes With ACR

ACR powder (J&K Scientific Chemicals, Shanghai) was dissolved in DMSO and then mixed evenly to form a concentrated storage solution. We diluted the concentrated storage solution of ACR to 2.5, 5, and 7.5 mM using M2 medium. Oocytes were exposed to the prepared ACR solution and cultured in a 37 °C incubator with 5% CO2 for 8.5 or 12 h. The concentration of ACR which had the most significant effect on the oocyte maturation rate was selected as the experimental concentration. Other experimental procedures required the control and experimental oocytes to be cultured for 8.5 h in a 37 °C, 5% CO2 incubator.

### Antibodies and Chemicals

Three antibodies were used for the current experiments: rabbit polyclonal anti-LAMP2 antibody (1:100) (no: 27823-1-AP, Proteintech, United States), rabbit polyclonal anti-Rab8A antibody (1:100) (ab188574, Abcam, UK), and rabbit polyclonal anti-Rps3 antibody (1:500) (ab181992, Abcam, UK), fluorescent anti-rabbit antibodies were purchased from Invitrogen (Carlsbad, CA, United States), and Hoechst 33342 and other reagents were purchased from Sigma Aldrich Corp.

### Immunofluorescence Staining and Confocal Microscopy

For the detection of Rps3, oocytes were incubated with 4% paraformaldehyde at room temperature for 30 min, then were transferred into permeable solution containing 0.5% Triton X-100 for 20 min, and again transferred to PBS solution containing 1% BSA for 1 h at room temperature. The oocytes were then transferred into Rps3 antibody solution and incubated overnight at 4 °C. For detecting LAMP2 (1:100) and Rab8A (1:100), the oocytes were exposed to 1% chain protease solution and incubated for 3 min to remove zona pellucida at 37 °C and 5% CO_2_ environment. After that, the oocytes were washed three times in M2 medium. After culturing for 12 h, we washed these oocytes three times with washing buffer (0.1% Tween 20 and 0.01% Triton X-100 in PBS) for 3 min each time and then incubated them in Alexa Fluor 488 goat anti-rabbit fluorescent antibody solution or Alexa Fluor 594 goat anti-rabbit fluorescent antibody solution (1:200) for 1 h at room temperature. The oocytes were incubated in Hoechst 33342 for 15 min at room temperature, fixed on a slide glass, and then observed using a laser confocal microscope (Zeiss LSM900, Germany).

### Mitochondria, ER, and Lysosome Detection in Living Oocytes

In order to detect the effects of ACR on mitochondria, endoplasmic reticulum, and lysosomes in oocytes, the oocytes were incubated in the miscible liquids of MitoTracker Red (1:600) (M7512, Eugene, OR, United States) or ER-Tracker Red (1:600) (C1014-1, Beyotime Biotechnology, Shanghai, China) or LysoTracker Red (1:3000) (C1046, Beyotime Biotechnology, Shanghai, China) and M2 culture medium for 30 min in the environment of 37 °C, 5% CO2. The oocytes were then washed once with M2 culture medium. Then, we observed each oocyte in the samples of experimental group and control group one by one under a laser confocal microscope.

### Golgi Apparatus Distribution Detection in Oocytes

For the purpose of detecting the distribution of Golgi apparatus in the oocytes, 1% chain protease was used to remove the zona pellucida at 37 °C, 5% CO_2_. The oocytes were incubated in miscible liquids of Golgi-Tracker Red (1:1) (C1043, Beyotime Biotechnology, Shanghai, China) and M2 medium at 4°C for 30 min and then washed with M2 medium at 4°C for 3 times. Afterward, the oocytes were incubated in miscible liquids of Hoechst 33342 and M2 culture solution at 37°C for 30 min. M2 culture solution was used to wash the oocytes once. Finally, each oocyte was detected under a laser confocal microscope.

### Statistical Analysis

Every detection index in the experiments was repeated more than three times, and over 15 oocytes were detected in both the control group and the experimental group in each experiment to minimize possible experimental errors in the results. The fluorescence results of the experiment were statistically analyzed by the GraphPad Prism Software (GraphPad, San Diego, CA). The statistical analysis of the results was conducted by using the t-test of two independent samples. The expression of experimental results was mean ± SD. A *p*-value less than 0.05 on one side can be considered statistically different.

## Results

### ACR Disrupts Polar Body Extrusion During Oocyte Maturation

**FIGURE 1:**
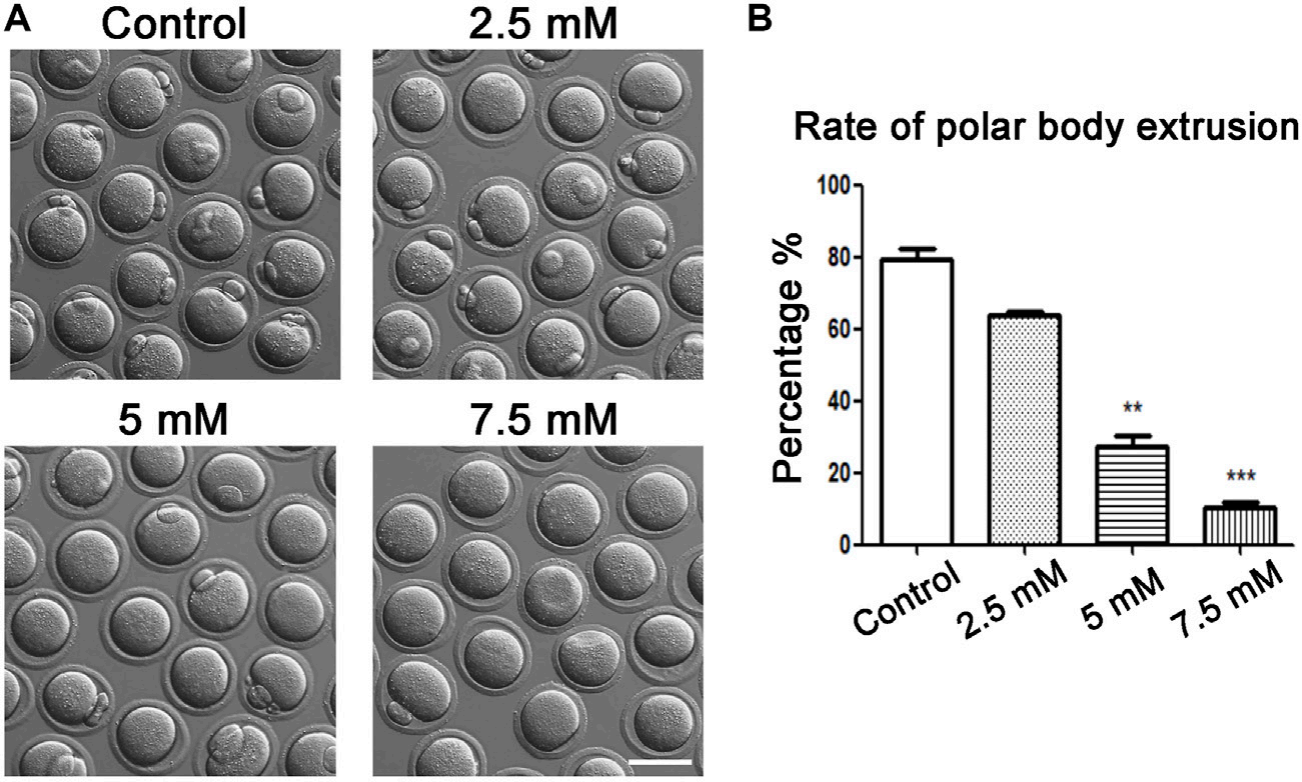
Toxic effects of ACR on oocyte maturation with different ACR dosages. **(A)** A typical picture of the first polar body in oocytes after ACR exposure. In the control group, most oocytes extruded the polar body, while there were few in the treatment groups. Bar = 80 μm. **(B)** The rate of the first polar body after ACR exposure in mouse oocytes. **p < 0.01, ***p < 0.001.

To explore the relative effective concentration of ACR on affecting oocyte maturation ability, four groups of oocytes were exposed to the concentration gradient of ACR solution at 0 mM, 2.5 mM, 5 mM, and 7.5 mM. As shown in Figure 1A, in the control group, almost all oocytes could mature and extrude the first polar body; compared with the control group, exposure to ACR inhibited oocyte maturation, and most oocytes were unable to extrude the first polar body. The statistical analysis data for the rate of polar body extrusion showed that the high-concentration ACR groups were remarkable than the control group in the 5 and 7.5 mM ACR groups (control, 79.47 ± 4.84%, n = 90 vs. 2.5 mM, 64.02 ± 0.88%, n = 84 vs. 5 mM, 27.54 ± 2.97%, *n* = 64 vs. 7.5 mM, 10.28 ± 1.39%, *n* = 45) (Figure 1B). Therefore, 5 mM was selected as the concentration of ACR in the following experiments.

### ACR Causes Mitochondrial Distribution and MMP Defects in Oocytes

**FIGURE 2:**
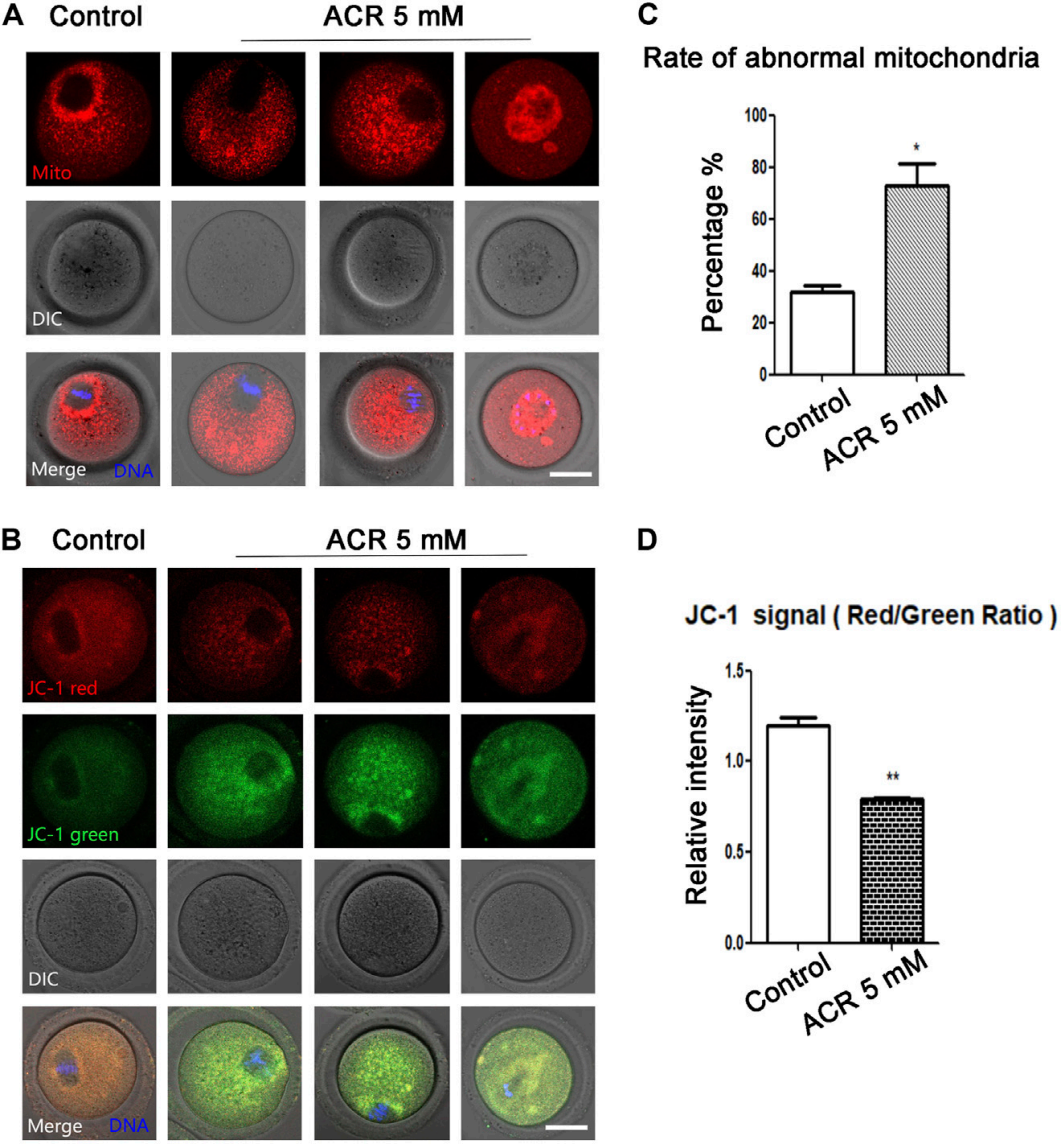
Toxic effect of ACR on mitochondria in mouse oocytes. **(A)** The typical picture of the distribution of mitochondria in oocytes after ACR exposure. In control oocytes, mitochondria accumulated at the spindle periphery but diffused in the treatment groups. Red, Mito-Tracker; blue, DNA. Bar = 30 μm. **(B)** The typical picture for Mitochondria-JC-1 red channel and green channel after ACR exposure in oocytes. Blue, DNA. Bar = 30 μm. **(C)** The abnormal rate of mitochondrial distribution in oocytes after ACR treatment. Histogram showed that the abnormal rate of mitochondria increased significantly in 5 mM ACR groups. **, *p* < 0.01. **(D)** The mitochondrial JC-1 signal ratio (red/green) after ACR treatment. The histogram showed that the ratio of fluorescence intensity of the JC-1 red/green channel was reduced in the 5 mM ACR group. **p < 0.01.

Since mitochondria play an important role in the process of cell energy metabolism, we first detected the effects of ACR on mitochondria-related functions of oocytes. First, Mito-Tracker was used to detect the distribution of mitochondria in oocytes. On the basis of fluorescence staining, in the control oocytes, mitochondria were accumulated at the spindle periphery area, while in the treatment group, the mitochondrial distribution was mainly scattered or accumulated into clumps in the cytoplasm (Figure 2A). It was apparent that the proportion of mitochondria surrounding the spindle in oocytes at MI stage was lower than that in the control group (control group: 31.65 ± 4.72%, *n* = 61; ACR group: 72.72 ± 15.1%, *n* = 52, *p* < 0.05) (Figure 2C). From the aforementioned results, it can be found ACR can affect the distribution of mitochondria in MI oocytes. In order to find out whether ACR also has toxic effects on the mitochondrial function of oocytes, we selected JC-1 as the membrane potential stain to detect whether the mitochondrial membrane potential of oocytes changed. As shown in Figure 2B, compared with the control group, the fluorescence intensity of the red channel of mitochondria-JC-1 in the ACR group decreased, while that of the green channel increased. We calculated the ratio of the red channel fluorescence intensity to the green channel fluorescence intensity in the control group and ACR group, and the data also confirmed our findings (control group: 1.2 ± 0.08, *n* = 56; ACR group: 0.79 ± 0.02, *n* = 53, *p* < 0.05) (Figure 2D). The results showed that ACR had toxic effects on the mitochondrial functions of oocytes.

### ACR Disturbs Rps3 and Endoplasmic Reticulum Accumulation in Oocytes

**FIGURE 3:**
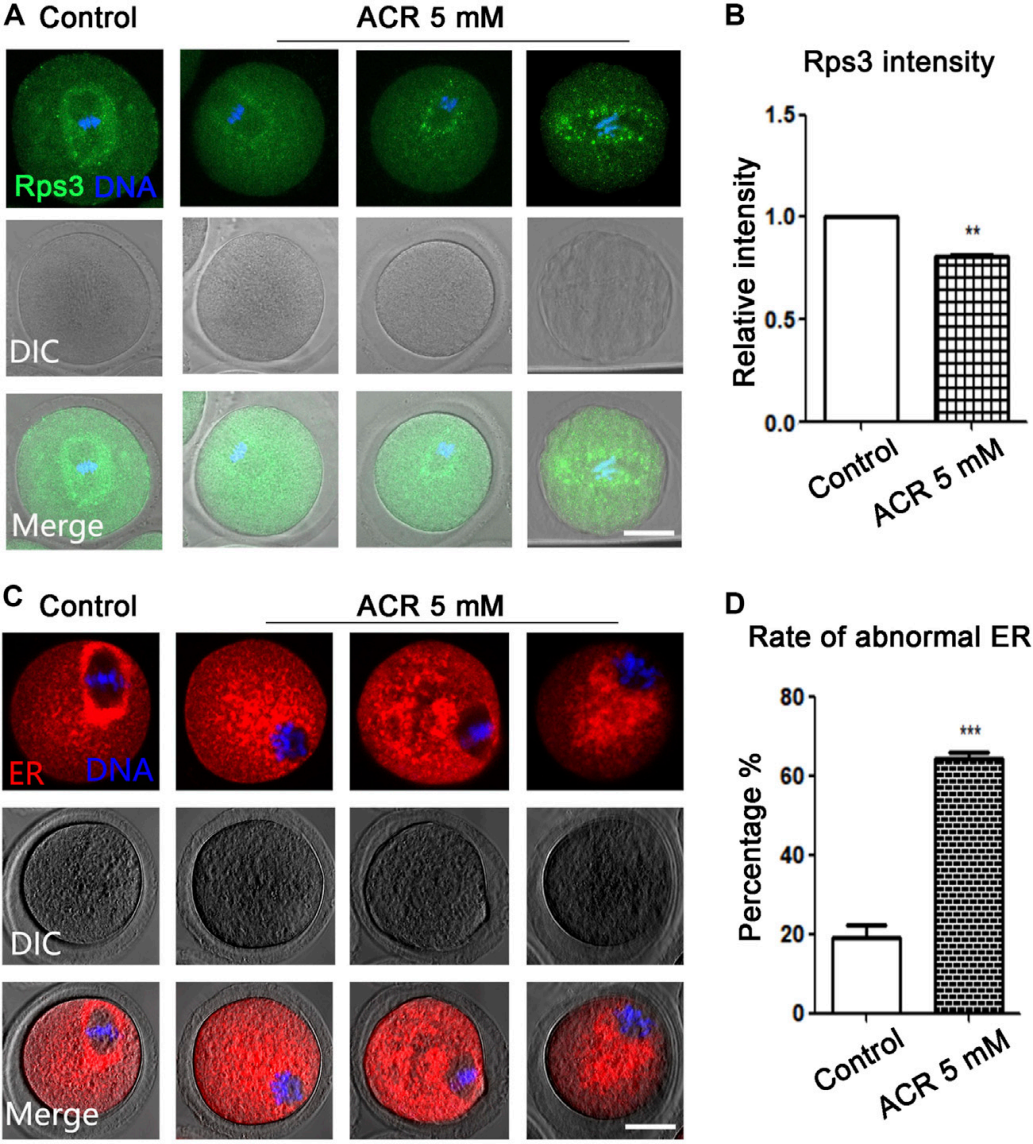
ACR decreases the distribution of ribosome protein Rps3 and ER in oocytes. **(A)** The typical fluorescence picture of ribosome protein Rps3 after ACR treatment. Rps3 diffused from the spindle periphery after ACR exposure compared with the control group. Green, Rps3; blue, DNA. Bar = 30 μm. **(B)** The fluorescence intensity of Rps3 after ACR exposure was significantly higher than that of the control group. *p < 0.05. **(C)** The typical picture for the distribution of ER after ACR treatment. Similar to ribosome, the ER diffused from the spindle periphery after ACR exposure compared with the control group. Red, ER-Tracker. Bar = 30 μm. **(D)** The abnormal rate of ER increased significantly in the 5 mM ACR group compared with the control group. ***p < 0.001.

Since the ribosome biosynthesis process requires energy participation, and ACR had toxic effects on the energy metabolism process of oocytes, we stained ribosome-labeling protein Rps3 in the control group and ACR group, respectively. We found that Rps3 localized near the meiotic spindle during oocyte maturation, while ACR exposure caused the diffusion of Rps3 at this area (Figure 3A). As shown in Figure 3B, based on the statistical analysis results, the fluorescence intensity of Rps3 in the ACR group was weaker than that of the control group in mouse oocytes (control group: 1.0, *n* = 112; ACR group: 0.86 ± 0.02, *n* = 102, *p* < 0.05). Endoplasmic reticulum is important for the mature process of protein in oocytes. Then, we used ER-Tracker to detect whether the endoplasmic reticulum distribution of MI oocytes was affected by ACR toxicity. As shown in Figure 3C, similar to Rps3, the distribution of the endoplasmic reticulum in MI oocytes was also at the spindle periphery area in the control group, while the distribution of endoplasmic reticulum in MI oocytes was also diffused into the cytoplasm in the ACR group, showing the abnormal phenotype of scattered or aggregated. According to the statistical results in Figure 3D, the abnormal rate of the endoplasmic reticulum in the control group is 19.25 ± 5.13% (*n* = 60), while that in the ACR group is 64.46 ± 2.67% (*n* = 54) (*p* < 0.05). Therefore, ACR had toxic effects on the function of ribosome protein Rps3 and endoplasmic reticulum in oocytes.

### ACR Affects Golgi Apparatus-Based Vesicle Transport in Oocytes

**FIGURE 4:**
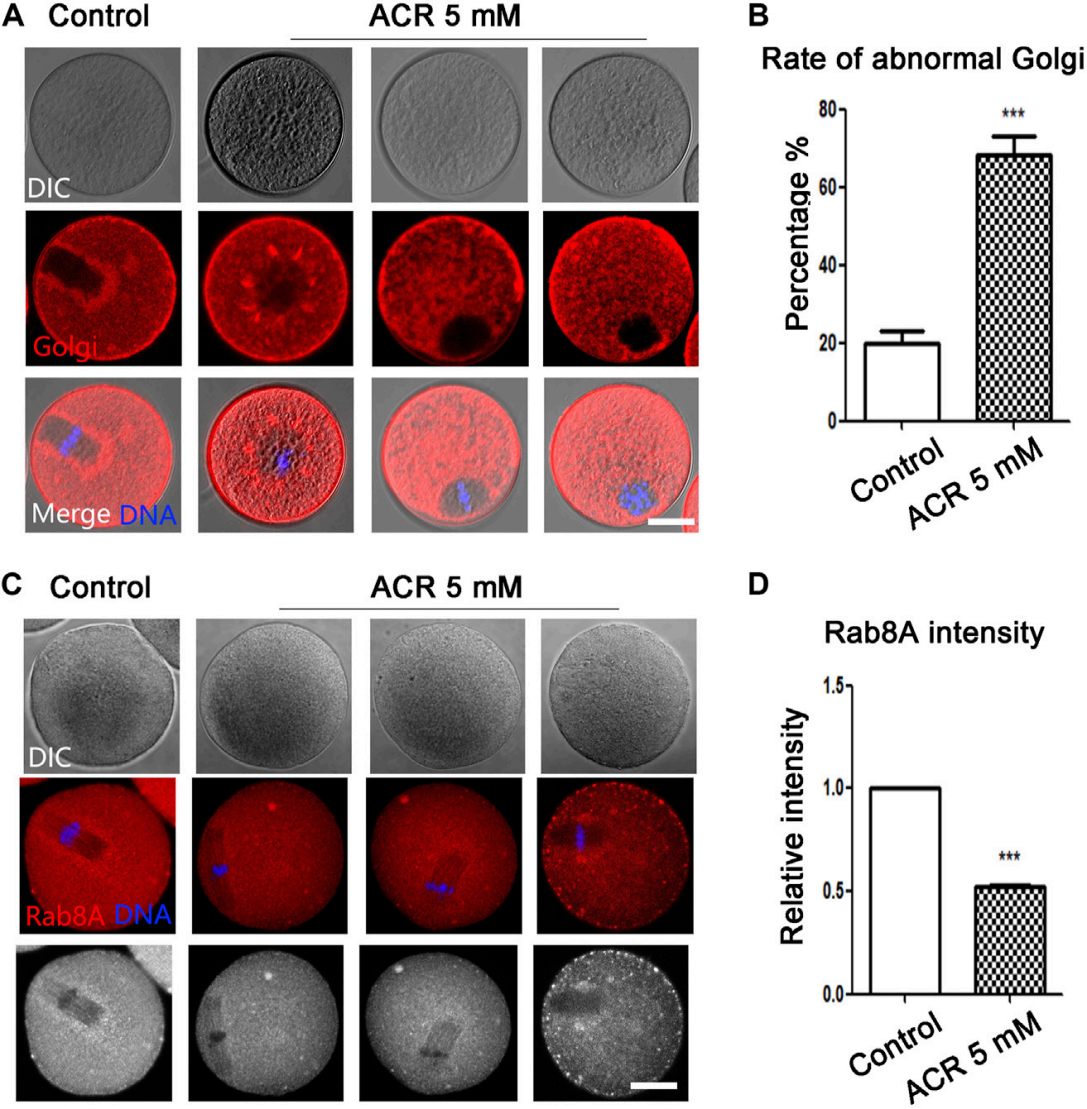
Toxic effects of ACR on the distribution and vesicle transport capacity of the Golgi apparatus in oocytes. **(A)** The typical picture of the distribution of the Golgi apparatus in oocytes after treatment. The Golgi apparatus fragment was observed in the ACR-exposed oocytes. Red, Golgi-Tracker; blue, DNA. Bar = 30 μm. **(B)** The abnormal rate of the Golgi apparatus increased significantly in 5 mM ACR groups. ***p < 0.001. **(C)** The typical fluorescence picture of Rab8A after ACR treatment. Few Rab8A signals were observed in the ACR-exposed oocytes. Red, Rab8A. Blue, DNA. Bar = 30 μm. **(D)** The fluorescence intensity of Rab8A after ACR exposure was significantly lower than that of the control group. *p < 0.05.

The Golgi apparatus plays an important role in the modification and transportation of protein. We next used Golgi-Tracker to detect the distribution of the Golgi apparatus. As shown in Figure 4A, the Golgi apparatus in the control group was distributed regularly around the spindle, but the distribution of the Golgi apparatus was scattered and irregular in oocytes in the ACR treatment group. According to the statistical analysis (Figure 4B), the abnormal rate of the Golgi apparatus in oocytes of the ACR treatment group was much higher than that in the control group (control group: 19.86 ± 5.59%, *n* = 52; ACR group: 68.36 ± 7.85%, *n* = 49, *p* < 0.05). We then detected the localization of vesicle transport protein Rab8A to judge the transport function of the Golgi apparatus. As shown in Figure 4C, the fluorescence signal of Rab8A in the control group mainly appeared around the spindle, while the Rab8A signal in the ACR group was mainly dispersed in the cytoplasm. As shown in Figure 4D, the fluorescence intensity of Rab8A in the ACR group was lower than that in the control group (control group: 1.0, *n* = 105; ACR group: 0.52 ± 0.02, *n* = 98, *p* < 0.05). These results showed that ACR could damage the distribution and function of the Golgi apparatus in oocytes.

### ACR Disrupts Lysosome Function in Mouse Oocytes

**FIGURE 5:**
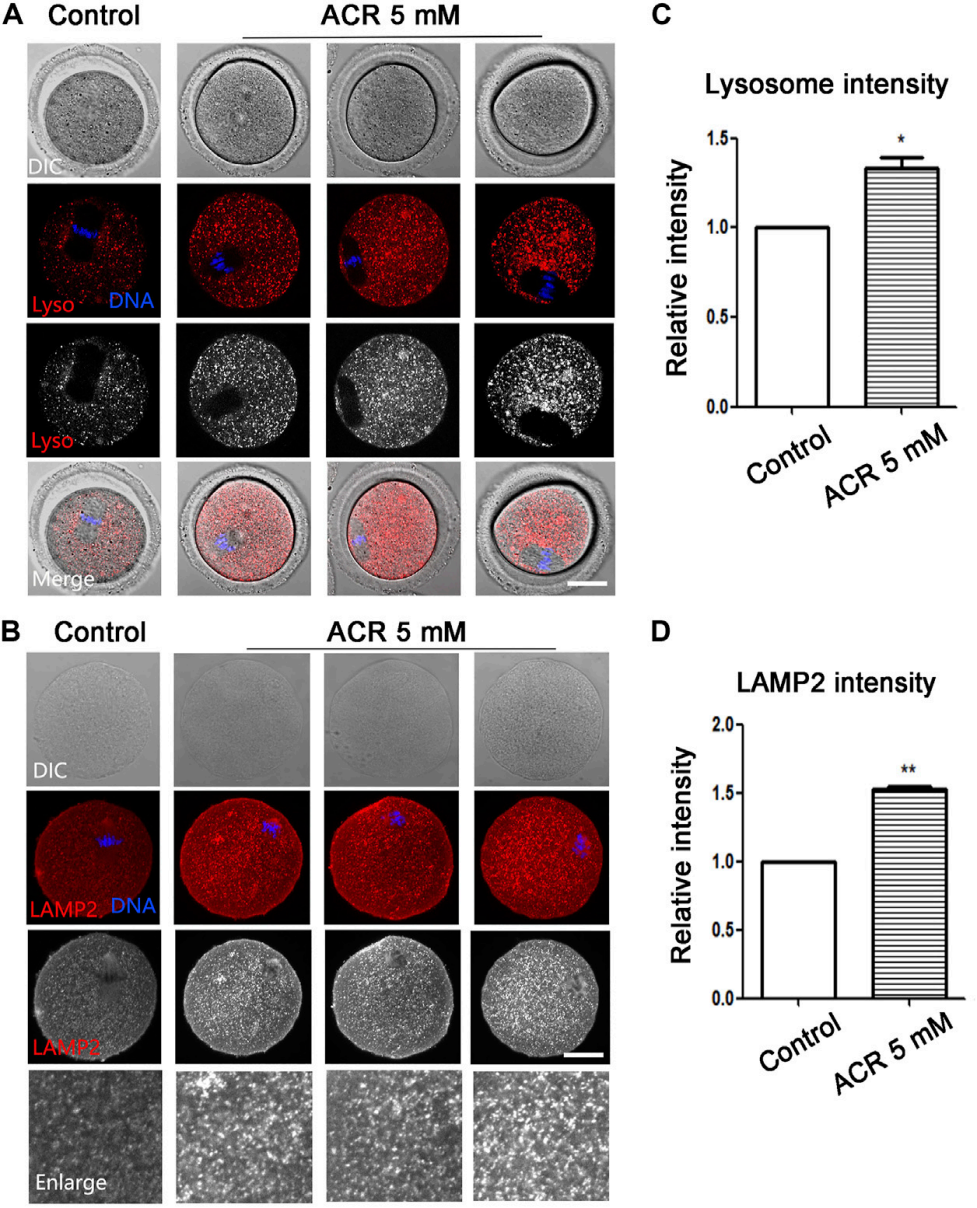
ACR causes the expression of lysosomes in mouse oocytes. **(A)** The typical fluorescence picture of lysosomes after ACR treatment. *p < 0.05. Red, Lyso-Tracker; blue, DNA. Bar = 30 μm. **(B)** The typical fluorescence picture of LAMP2 after ACR treatment. Red, LAMP2; blue, DNA. Bar = 30 μm. **(C)** The fluorescence intensity of lysosomes after ACR exposure was significantly higher than that of the control group. *, *p* < 0.05. **(D)** Compared with the control group, the fluorescence intensity of LAMP2 in oocytes decreased after ACR exposure. **p < 0.01.

The lysosome is the organelle involved in protein degradation and digestion in the oocytes. Lyso-Tracker was used to detect the fluorescence intensity of lysosomes. As shown in Figure 5A, there were barely any signals of lysosomes in the cytoplasm of oocytes in the control group. However, the signals of lysosomes of oocytes in the ACR group were much stronger. According to statistical analysis, the fluorescence intensity in the treatment group was much higher than that in the control group (control group: 1.0, *n* = 61; ACR group: 1.33 ± 0.10, *n* = 43, *p* < 0.05) (Figure 5C). To further analyze the effect of ACR on oocyte lysosomes, LAMP2 was used to examine the status of the lysosome membrane. Similar to Lyso-Tracker staining, there was low expression of LAMP2 in the control oocytes, while strong fluorescence signals were observed in the treatment group (Figure 5B). The statistical analysis data showed that compared with the control group, the fluorescence signals of LAMP2 in oocytes of the ACR group are much higher (control group: 1.0, *n* = 89; ACR group: 1.53 ± 0.03, *n* = 87, *p* < 0.01) (Figure 5D). The results revealed that ACR could destroy the function of lysosomes in oocytes.

## Discussion

In the present study, we detected the distribution and function of organelles in the oocytes under ACR exposure. We examined the mitochondria, endoplasmic reticulum, and Golgi apparatus, ribosomes, and lysosomes to assess the damage of ACR exposure to oocyte quality *in vitro*. Our results provided the explanation for the toxicity of ACR on the quality of oocytes in mice.

Previous results showed that ACR had toxic effects on the reproductive system of female mice, indicating that ACR can cause the decreased first polar body excretion rate of oocytes in the *in vivo* model, an important index to judge the nuclear maturity of oocytes (Liu et al., 2015). However, the effective doses in the *in vitro* environment are still unclear, and the causes for the decline in oocyte maturation competence under ACR exposure are not reported. Our results showed that 5 mM ACR could effectively reduce the maturation rate of oocytes *in vitro*. To demonstrate the damage of ACR to oocyte quality from the perspective of organelles, we first examined the distribution and function of mitochondria, the energy-producing organelles. We found that ACR could lead to abnormal mitochondrial distribution and decreased mitochondrial membrane potential in mouse oocytes, indicating that ACR could disrupt the energy metabolism process of oocytes. This is similar to the previous results, showing that ACR can lead to the disappearance of mitochondrial membrane potential in human lymphocytes (Salimi et al., 2021). ACR also can induce the decrease in the mitochondrial membrane potential of IEC-6 cells through the pathway of cytochrome C content alteration and caspase-3 activation (Zhang et al., 2017).

Due to the fact that ribosome is the only organelle that translates mRNA into polypeptide chains in cells (Catez et al., 2018), protein synthesis requires large amounts of energy (Morita et al., 2015). We examined ribosome protein Rps3 and found that ACR could lead to abnormal Rps3 localization in mouse oocytes. Using a variety of omics methods to detect ACR-contaminated *Saccharomyces cerevisiae*, it can be realized that ribosome function is damaged, protein synthesis decreased, and protein consumption increased, which will lead to autophagy initiation and yeast poisoning (Zhen et al., 2021). Thus, ribosome aging could lead to the low fertilization rate of mouse oocytes, and this could be rescued by melatonin supplement (Tamura et al., 2017). Our results indicated that ACR had similar effects on lysosomes in the mammalian oocyte model. Endoplasmic reticulum (ER) is a membranous organelle surrounding the nucleus which modifies the new polypeptide chain and transfers it to the Golgi apparatus for further maturation (Stevenson et al., 2016). The membrane of the ER and mitochondria can form a common structural region—mitochondria-associated membranes (MAMs) (Rieusset, 2018). Therefore, we detected the effect of ACR on the distribution of ER in the oocytes, and we showed that similar to ribosome protein Rps3, ER localization was also disrupted, indicating that ACR also had toxic effects on the function of ER in oocytes. The swelling of ER in cerebellar neurons can be observed when exposed to ACR on rats, accompanied by the increased expression of stress-related proteins in the endoplasmic reticulum for the muscle strength and motor coordination ability of rats (Yw et al., 2021). The addition of tauroursodeoxycholic acid to bovine cumulus–oocyte complex can inhibit the expression level endoplasmic reticulum stress-related proteins and apoptosis-related genes in bovine oocytes, thus improving the quality of oocytes and the development rate of blastocysts (Khatun et al., 2020). Therefore, ACR could affect protein synthesis and modification by disrupting the lysosome and ER in oocytes.

The Golgi apparatus is a polar organelle composed of flattened membrane sacs, small vesicles, and large vesicles, which help the immature proteins transferred from the ER to modify and fold to form functional proteins (Stalder and Gershlick, 2020). We detected the distribution of the Golgi apparatus and the expression of vesicle transporter Rab8A in oocytes, and we found that ACR could lead to abnormal Golgi apparatus distribution and decreased expression of Rab8A in mouse oocytes. Previous studies have also shown that cell exposure to different microenvironments can cause abnormal distribution and function of the Golgi apparatus. For example, exposure to zearalenone leads to the abnormal distribution of the Golgi apparatus in porcine oocytes, which causes the decrease in the maturation rate of porcine oocytes and affects the quality of porcine oocytes (Wang et al., 2021). Podophyllotoxin can cause the Golgi apparatus fragment of mouse oocytes and vesicle transport defects, thus causing the meiosis failure of mouse oocytes (Lu et al., 2021). Lysosomes are round organelles formed by a monolayer covering various acid hydrolases (Lie and Nixon, 2018), which mainly participate in the metabolic activities of various substances in cells (Albanese et al., 2019). We examined whether ACR also had adverse effects on the function of lysosomes, and we found that lysosome signals and the marker protein LAMP2 all increased under ACR exposure in oocytes. Other factors in the environment also affect normal lysosome function. For example, citrinin can lead to increased expression of LAMP2 and other autophagy-related genes in mouse oocytes, thereby reducing oocyte quality (Sun et al., 2020). The addition of high-concentration ACR to the diet of *Theba pisana* can cause the stability of lysosomal membrane to decrease and the indexes related to oxidative stress to increase after 14 days, thus inducing cell death (Radwan et al., 2019). Together with our data, we showed that ACR exposure could affect protein degradation and metabolic activities in oocytes.

In summary, our results showed that ACR could cause abnormal functions of organelles such as the mitochondria, ribosome, ER, and Golgi apparatus in mouse oocytes, which affected protein synthesis, modification, and transport, thereby reducing the quality of oocytes.

## Data Availability

The original contributions presented in the study are included in the article/Supplementary Material, further inquiries can be directed to the corresponding authors.
